# A Novel Triphenylamine-Based Flavonoid Fluorescent Probe with High Selectivity for Uranyl in Acid and High Water Systems

**DOI:** 10.3390/s22186987

**Published:** 2022-09-15

**Authors:** Bing Liu, Wenbin Cui, Jianliang Zhou, Hongqing Wang

**Affiliations:** 1 Library, University of South China, Hengyang 421001, China; 2 School of Chemistry and Chemical Engineering, University of South China, Hengyang 421001, China; 3 School of Nuclear Science and Technology, University of South China, Hengyang 421001, China

**Keywords:** UO_2_^2+^, triphenylamine, flavonoid, turn off, fluorescent probe

## Abstract

Developing a fluorescent probe for UO_2_^2+^, which is resistant to interference from other ions such as Cu^2+^ and can be applied in acidic and high-water systems, has been a major challenge. In this study, a “turn-off” fluorescent probe for triamine-modified flavonoid derivatives, 2-triphenylamine-3-hydroxy-4H-chromen-4-one (abbreviated to HTPAF), was synthesized. In the solvent system of dimethyl sulfoxide:H_2_O (abbreviated to DMSO:H_2_O) (*v*/*v* = 5:95 pH = 4.5), the HTPAF solution was excited with 364 nm light and showed a strong fluorescence emission peak at 474 nm with a Stokes shift of 110 nm. After the addition of UO_2_^2+^, the fluorescence at 474 nm was quenched. More importantly, there was no interference in the presence of metal ions (Pb^2+^, Cd^2+^, Cr^3+^, Fe^3+^, Co^2+^, Th^4+^, La^3+^, etc.), especially Cu^2+^ and Al^3+^. It is worth noting that the theoretical model for the binding of UO_2_^2+^ to HTPAF was derived by more detailed density functional theory (DFT) calculations in this study, while the coordination mode was further verified using HRMS, FT-IR and ^1^HNMR, demonstrating a coordination ratio of 1:2. In addition, the corresponding photo-induced electron transfer (PET) fluorescence quenching mechanism was also proposed.

## 1. Introduction

Currently, nuclear energy is an important component of non-conventional energy sources, and nuclear energy development has become a key factor in the energy transition of countries [1]. Uranium is an important fuel for nuclear energy, but due to its inherent radioactivity and toxicity, the vigorous development of nuclear energy can also pose a serious threat to the environment at the same time [2]. For example, a large amount of uranium-containing acidic wastewater from uranium processing is more or less inevitably discharged into the surrounding environment [3]. Moreover, uranium exists mainly as UO_2_^2+^ species in an acidic environment, which is highly migratory in water and contaminates the surrounding soil and water bodies [4,5]. Hence, the effective detection of UO_2_^2+^ under acidic conditions is of great interest for pollution control. In recent years, there are many methods for the detection of uranyl ions, such as voltammetry [6,7], surface-enhanced Raman spectroscopy [8,9], and electrochemical methods [10,11], but these instrumental methods usually require high costs, complex instruments, and time-consuming calibration, which limit the scope of application [12].

Compared to these methods, fluorescence methods are widely used due to their low cost, fast response, and naked-eye recognition [13]. Therefore, the research of fluorescence detection of uranyl has become a current research hotspot [14,15,16]. However, one problem with the detection of uranyl ions by fluorescence is the presence of interference from other metal ions, especially Cu^2+^ and Al^3+^, and most solutions to this problem are the addition of masking agents [13,17,18]. Another problem is that high water systems are prone to quenching (ACQ) effects due to aggregation of organic fluorophores, leading to inaccurate fluorescence detection [19]. To solve this problem, many reports have chosen to add a large amount of organic solvent to the system to improve the detection efficiency [20,21], but this is not favorable for application in real water samples. Therefore, it is critical to develop a highly selective fluorescence method for uranyl detection in high-water systems.

As well as the above-mentioned organic solvents that can reduce the ACQ effect, the aggregation-induced emission enhancement (AIEE) effect can also be exploited [22,23]. AIEE properties can be introduced into organic fluorescent dyes by conjugation to fluorophores with specific nonplanar structures, which effectively prevents π-π stacking of aromatic rings in the fluorophores [24]. In other words, it does not stack easily in organic solvents and cannot be aggregated to induce emission enhancement, while easy π-π stacking in high water systems makes fluorescence emission enhancement. As a result, the AIEE property can help fluorescent probes to maintain their strong fluorescence properties even in the aggregated state or in undesirable solvents (e.g., water), thus enabling the sensitive detection of targets in water [25]. Triphenylamine is a non-planar structured fluorophore due to its single bond rotatability [26], which can be designed with other organic groups to introduce AIEE effect and thus reduce the ACQ effect [27,28]. Moreover, triphenylamine-based fluorescent molecules are usually characterized by high fluorescence quantum yields and good photostability [29,30].

**Scheme 1:**
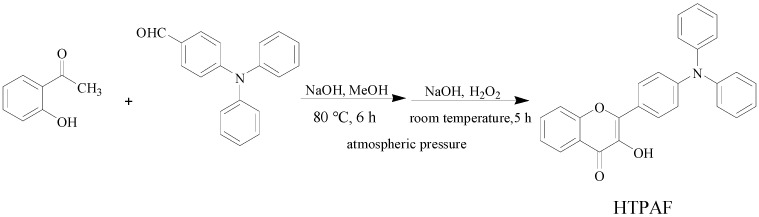
Synthesis route of HTPAF.

Flavonoid dyes are widely used to prepare fluorophores for various spectroscopic probes due to their excellent photophysical properties, and flavonoid-based fluorophores usually emit efficient and persistent fluorescence with large Stokes shifts, which minimizes self-absorption and reduces background interference [31,32,33]. Moreover, flavonoid derivatives also have the advantages of simple synthesis and easy introduction of functional groups [34]. Over the years, many flavonoid-based fluorescent probes have been developed for the detection of various analytes, including metal ions, anions, and reactive species [35]. Here, our team was inspired to develop a fluorescent probe, HTPAF (Scheme 1), by specific modification of flavonoids in this study, which has a large Stokes shift and can be highly selective for uranyl ions in acidic and high water ratios without the interference of other metal ions.

## 2. Experimental Section

### 2.1. Reagents and Instrumentation

All chemical reagents are of analytical purity grade, which were purchased on Aladdin and Maclean’s platforms and could be used without purification. Among them, UO_2_^2+^, Ni^2+^, Bi^3+^, Ca^2+^, K^+^, Mg^2+^, Cr^3+^, Fe^3+^, Zn^2+^, Cu^2+^, In^2+^, Pb^2+^, Al^3+^, Nd^2+^, Eu^3+^, Na^+^, Cd^2+^, Zr^4+^, Sr^2+^, Dy^3+^, Sm^3+^, Th^4+^, La^3+^, Ag^+^, and Co^2+^ plasma solutions were prepared by dissolving nitrates of different metals in deionized water. A quartz cuvette (GB/T26791-2011 and Q/320223, Silver Star Analytical Experimental Devices Factory, made in Yixing, China), an automatic potentiometric titrator (ZDJ-3D, made in Beijing, China), a tabletop high-speed refrigerated centrifuge (Sigma/3k15, made in Germany), a freeze dryer (scientz-12, made in Ningbo, China), a nuclear magnetic resonance spectroscopy (Bruker 600-MHz, made in USA), a UV-visible spectrophotometer (UV-Vis 3900, made in Japan), a rotary evaporator (RE-52AA, made in Shanghai, China), an infrared spectroscopy (IRPrestige-21 made in Japan), a fluorescence spectrometer (Hitachi F-7000, made in Japan), a nuclear magnetic resonance hydrogen spectroscopy (Bruker 500 MHz, made in USA), and a high-resolution mass spectrometry (Thermo Scientific Q Exactive, made in USA) were used.

### 2.2. Synthesis Procedure

#### 2.2.1. Synthesis of HTPAF

The synthetic route of HTPAF was inspired by the literature [25], and the synthesis process was shown in Scheme 1.

Added to a 100 mL round bottom flask were 2-Hydroxyacetophenone (1.075 g, 5 mmol), 4-Diphenylamino-benzaldehyde (1.366 g, 5 mmol), sodium hydroxide (0.9 g), and methanol solution (30 mL). The mixture solution was refluxed for 6 h, cooled to room temperature, and then stirred overnight. Next, NaOH solution (0.5 M, 30 mL) and H_2_O_2_ (30%, 4 mL) were added and the reaction was stirred at room temperature for 5 h. After that, the solution was diluted in a beaker with 300 mL of ice water, the pH of the solution was adjusted to 6 with concentrated hydrochloric acid, ethyl acetate was extracted, anhydrous Na_2_SO_4_ was added to remove the water from the organic layer, filtered, and the organic solvent was distilled off under reduced pressure using a rotary evaporator to obtain a yellow solid product. The solid product was freeze-dried in a freeze dryer to obtain the final product. Yield: 45.6%, melting point: 103.6–106.4 °C. FT-IR (KBr): 3327, 3077, 1686, 1588, 898, 756 cm^−1^. ^1^H NMR (400 MHz, DMSO-d6) δ (ppm): δ 7.78–7.68 (m, 3H), 9.77 (s, 1H), 7.42 (t, J = 7.71 Hz, 5H), 7.28–7.15 (m, 9H), 6.95–6.84 (m, 3H). ^13^CNMR (100 MHz, DMSO-d6) δ(ppm): δ 179.27, 153.69, 152.56, 143.48, 135.21, 127.81, 126.82, 125.97, 125.55, 124.99, 124.00, 120.78, 120.32. HRMS (ESI) m/z: theoretical value (C_27_H_21_NO_3_): 407.15 (M^+^); found(M^+^): 407.1470, found(M+Na)^+^: 430.1358 (Figure S3).

#### 2.2.2. Synthesis of HTPAF+UO_2_^2+^

**Scheme 2:**
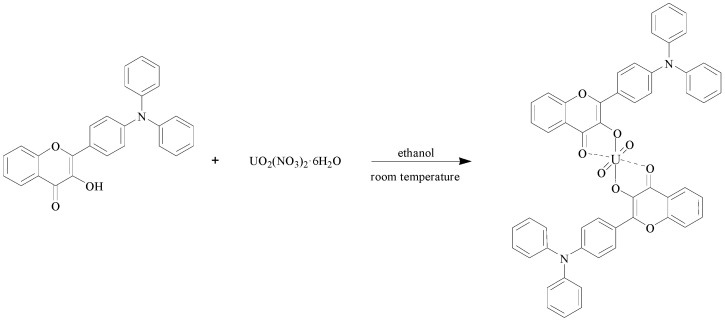
Synthesis route of HTPAF+UO_2_^2+^.

The formation process of uranyl ion and fluorescent probe HTPAF ligand compounds was shown in Scheme 2.

An amount of 0.5 mmol (0.204 g) of HTPAF compound was dissolved in 30 mL of ethanol and stirred for 30 min at room temperature. Then 2.2 mmol (0.151 g) of uranyl nitrate hexahydrate was accurately weighed and dissolved in 40 mL of ethanol, and then the uranyl solution was added dropwise to the HTPAF solution and reacted for 3–4 h. Rotary evaporation, repeated washing with deionized water, and low temperature recrystallization in anhydrous ethanol/water system resulted in a reddish-brown solid powder. The yield was 24%, FT-IR (KBr): 3410, 3035, 1689, 1585, 1506, 1489, 1284, 925, 825 cm^−1^. HRMS theoretical value (UC_54_H_40_N_2_O_8_): 1082.93 ([M-H]^−^: 1081.93; actual value: [M-H]^−^: 1079.3284).

### 2.3. Spectroscopic Analysis Procedure

HTPAF (250 μL, 1 × 10^−3^ M) was added to a 10 mL colorimetric tube containing UO_2_^2+^ ions (250 μL, 1 × 10^−3^ M). Then, the solvent system was made to dilute to the mark (DMSO:H_2_O, *v*/*v* = 5:95, pH = 4.5). The solution was shaken for 30 s to mix well and waited for 5 min at room temperature, then some of the solution was transferred to a 5 mL cuvette and the fluorescence intensity and absorbance of the solution were tested by fluorescence spectrometer and UV spectrometer. The parameters of the machine for testing fluorescence spectra were set: the excitation wavelength was 364.0 nm, the width of the emission slit and the excitation slit were 5 nm and 10 nm, respectively, and the voltage was 500 volts.

### 2.4. Determination of Dissociation Constants

The electrode system was calibrated at three points to pH = 4.01, 6.86, and 9.18 using standard buffer solutions. An amount of 1 × 10^−3^ mol/L HTPAF and 0.1 mol/L HCl were added sequentially to the stirrer in corresponding amounts, and then the HTPAF solution was diluted with mixed solvent (DMSO:H_2_O, *v*/*v* = 5:95, pH = 4.5) at a concentration of 1 × 10^−5^ mol/L. Next, a 0.1 mol/L basic solution of sodium hydroxide was prepared using a solvent system of DMSO:H_2_O = 5:95 (*v*/*v*, pH = 4.5) and the burette was washed three times with sodium hydroxide solution and then filled with the basic solution. Then, the change in the real-time potential value was detected by adding 0.1 mol/L 0.05 mL of alkaline solution to the stirrer each time. A pH meter with high accuracy was also used to note down the pH data of the instantaneous potentials. The average value was obtained from the data of three measurements, and finally, the data were entered into a PC workstation to calculate the acid dissociation constant of HTPAF using a super-square.

### 2.5. Theoretical Calculation Parameter Settings

All calculations concerning quantum chemistry in this study were done by Gaussian 09 software. In order to more closely simulate the uranium elements in the real environment, a smaller nuclear ECP/ECP60MWB pseudopotential basis set considering relativistic effects was used [36,37,38]. For the elemental setup, the conventional B3LYP/6-311G(d,p) basis set was used, and in addition, the involvement of van der Waals forces in the system was added by Grimme’s D3 method, and the interaction of DMSO with water was simulated by the solvation model density (abbreviated to SMD) method under the isoelectric focusing polarizable-continuum model (abbreviated to IEF-PCM) rules [39,40,41,42].

## 3. Results and Discussion

### 3.1. Effect of Different Solvents on HTPAF

**Figure 1:**
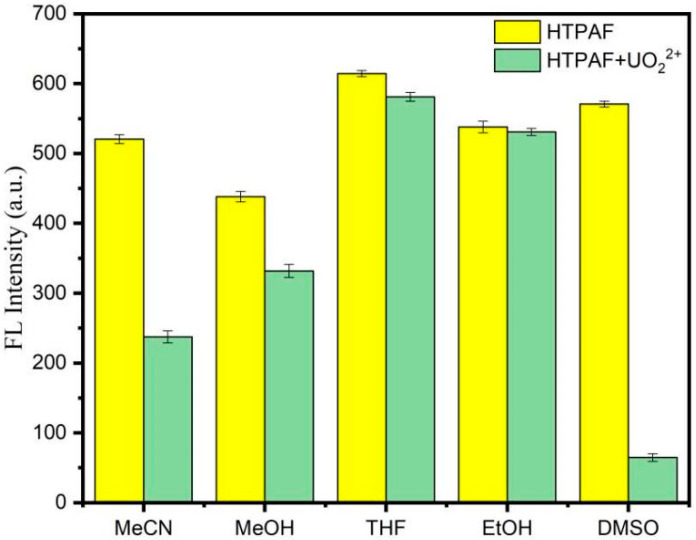
Fluorescence intensity of HTPAF and HTPAF+UO_2_^2+^ systems in various organic solvents (MeCN, MeOH, THF, EtOH, and DMSO) (HTPAF, 50 μM, UO_2_^2+^, 50 μM).

The solvent has a significant impact on the fluorescence performance of the probe [43], so the fluorescence intensity of the fluorescent probe HTPAF was studied in the presence and absence of UO_2_^2+^ in various organic solvents (acetonitrile, methanol, tetrahydrofuran, ethanol, and dimethyl sulfoxide) (abbreviated to MeCN, MeOH, THF, EtOH, and DMSO) (Figure 1). Comparing with different organic solvents, it is known that the more pronounced quenching effect was from the HTPAF+UO_2_^2+^ system in DMSO. Therefore, DMSO was chosen as the organic solvent for the subsequent experiments.

### 3.2. Influence of Solvent Ratio on HTPAF

**Figure 2:**
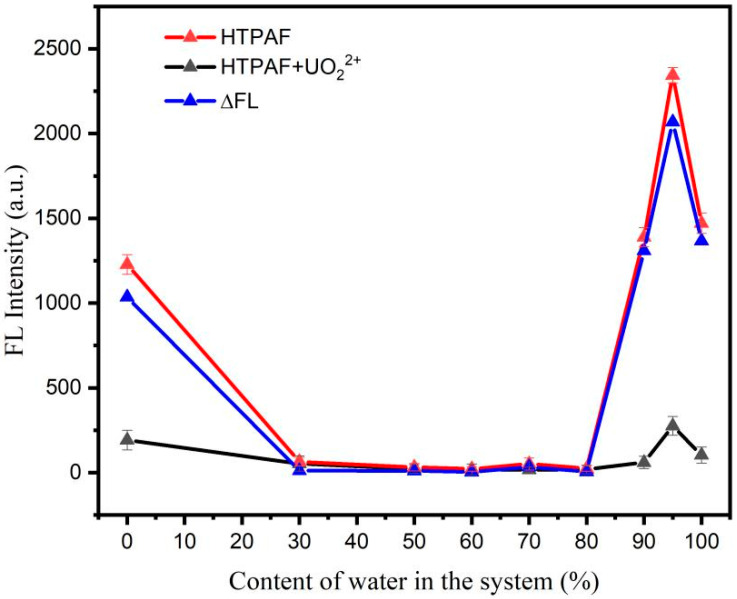
In the solvent system of DMSO:H_2_O, the fluorescence changes of HTPAF(50 μM) fluorescent probe before and after adding UO_2_^2+^ under different water ratios.

As can be seen in Figure 2, the fluorescent probe HTPAF was fluorescent in pure organic solvent, and when the proportion of water in the solvent system started to increase, from 10% to 70%, the fluorescence intensity of the solution system decreased sharply. The reason was that in the solvent system with a large proportion of organic solvents, HTPAF was in the dissolved state rather than in the stacked state. At this time, the triphenylamine structure in HTPAF was easily rotated in this system. When excited by light, the energy was released in the form of rotation and vibration rather than in the form of emitted light [44], so the fluorescence intensity of HTPAF was low at this time. However, when the proportion of water in the solvent system was increased to 90%, the fluorescence intensity of the probe increased exponentially because the fluorescence aggregation-induced luminescence enhancement effect (AIEE) began to appear for the fluorescent probe with the triphenylamine group, which made it easier to identify the fluorescence quenching of the HTPAF probe with the naked eye under UV light after the addition of uranyl ions. The fluorescence intensity of HTPAF reached its highest value when the proportion of water was continued to increase to 95%, and continued to increase to 100%, the fluorescence intensity gradually diminished. Therefore, the solvent system of DMSO:H_2_O = 5:95 was chosen for the subsequent fluorescence testing experiments.

### 3.3. Effect of pH on the Detection of UO_2_^2+^

**Figure 3:**
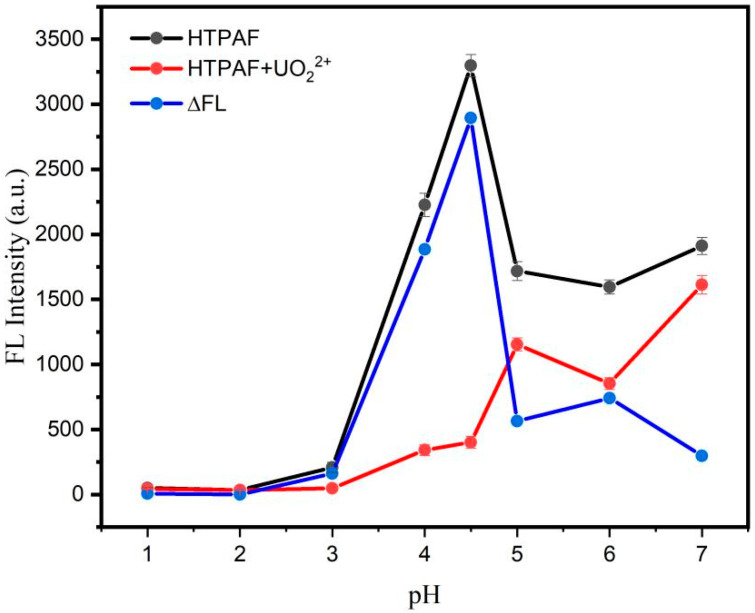
Fluorescence intensity of HTPAF before and after the addition of UO_2_^2+^ at different pH and the fluorescence change values (HTPAF, 50 μM, UO_2_^2+^, 50 μM).

**Figure 4:**
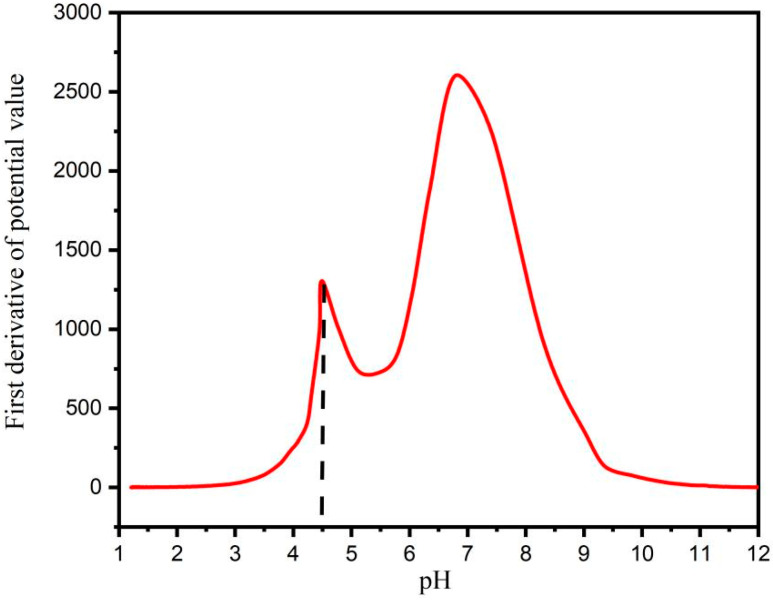
The first derivative of the fluorescent probe HTPAF solution (containing 0.1 mol/L HCl) titrated with 0.1 mol/L NaOH.

**Figure 5:**
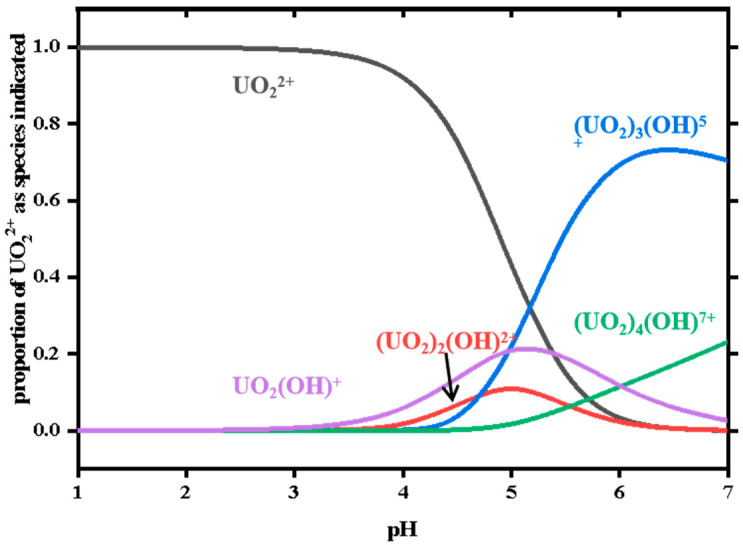
Ref. [17] Species distribution of uranyl ions at different pH.

As shown in Figure 3, the fluorescent probe HTPAF showed a strong fluorescence at pH 4.0–7.0. However, after the addition of UO_2_^2+^ ions, there was a strong fluorescence quenching at pH = 4.5. In order to clarify the fluorescence intensity change, the fluorescence intensity change values (ΔFL) before and after the addition of uranyl ions were indicated in the figure. The fluorescence change values clearly showed an increase at pH 1.0–4.5 and a sharp decrease at pH 4.5–5.0, followed by a slight rise and fall after pH 5.0. Therefore, the most pronounced fluorescence change and the highest degree of quenching were observed at pH = 4.5. According to the potentiometric titration (Figure 4) and the analysis of the results calculated by Visual MINTEQ software (Figure 5) [17], the main species of HTPAF and uranium were positively charged at pH < 4.0 and easily protonated, which made them difficult to bind. While the pH was in the range of 4.5–6.0, the main species of HTPAF was HTPAF^-^, but the main species of uranium was still UO_2_^2+^ (pH = 4.5–5.0), and the electrostatic interaction would promote the binding of the probe to uranium. It is worth noting that when pH > 5.0, uranyl gradually started to hydrolyze and produce precipitation, which was not favorable for detection. More importantly, considering the species state of the probe and uranium as well as the final detection effect, especially pH = 4.5 was more acidic and more suitable for practical applications compared to pH = 5.0. Therefore, pH = 4.5 was used as the assay condition in this study.

### 3.4. Selectivity and Competitiveness

**Figure 6:**
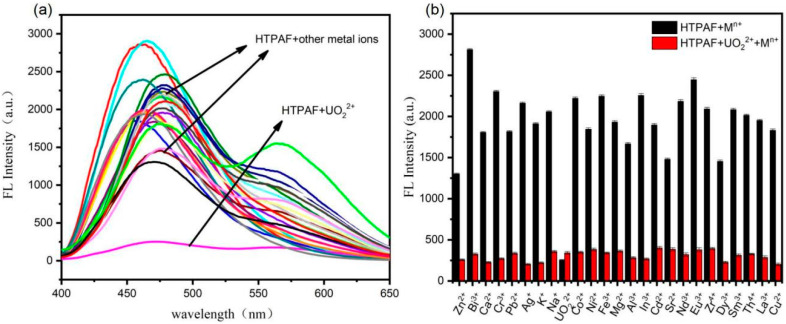
(**a**) Changes in fluorescence intensity of fluorescent probe HTPAF (10 μM) when an equivalent amount of metal ions (10 μM) was added (**b**) HTPAF (10 μM) versus UO_2_^2+^ (10 μM) (DMSO:H_2_O = 5:95 *v*/*v* and pH = 4.5) selectivity and anti-interference.

In order to investigate the fluorescence recognition selectivity of HTPAF for UO_2_^2+^ under acidic conditions, equal amounts of different metal ions and uranyl ions (Ni^2+^,Bi^3+^,Ca^2+^, K^+^, Mg^2+^, Cr^3+^, Fe^3+^, Zn^2+^,Cu^2+^, In^2+^, Pb^2+^, Al^3+^, Nd^2+^, Eu^3+^, Na^+^, Cd^2+^, Zr^4+^, Sr^2+^, Dy^3+^, Sm^3+^, Th^4+^, La^3+^, Ag^+^, Co^2+^, and UO_2_^2+^) were added to the solvent system of DMSO:H_2_O (*v*:*v* = 5:95, pH = 4.5), respectively, and the corresponding fluorescence spectra were obtained by excitation at 364 nm to determine the selectivity of HTPAF for UO_2_^2+^, and the results were shown in Figure 6a. As can be judged from Figure 6a, HTPAF did not respond to the most common metal ions except UO_2_^2+^. In other words, the HTPAF fluorescent probe designed in this study could achieve specific recognition of UO_2_^2+^ in the presence of most metal ions, especially Cu^2+^. Meanwhile, competition experiments were performed by adding UO_2_^2+^ to the HTPAF solution in the presence of other metal ions (Figure 6b). As could be seen in Figure 6b, most of the competing cations did not interfere significantly with the detection of UO_2_^2+^ by HTPAF. Therefore, the designed HTPAF fluorescent probe detected UO_2_^2+^ with relatively high interference resistance.

### 3.5. Fluorescence Titration Experiment of HTPAF on UO_2_^2+^ and Determination of Sensitivity

**Figure 7:**
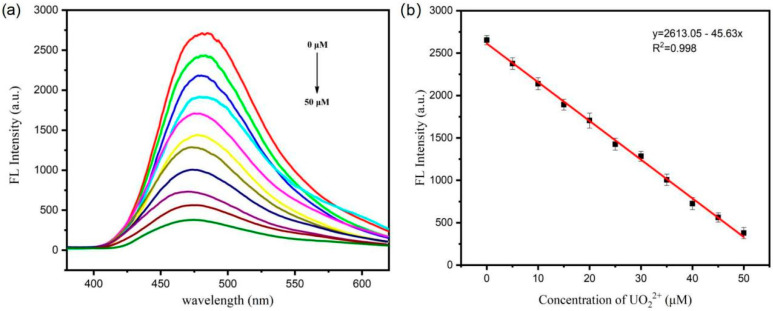
(**a**) Fluorescence intensity change when adding 0–50 μM of UO_2_^2+^ to HTPAF solution (**b**) Correlation between HTPAF fluorescence intensity and UO_2_^2+^ concentration (0–50 μM).

As shown in Figure 7a, when the concentration of UO_2_^2+^ was in the range of 0–50 μM, the fluorescence of the HTPAF solution system gradually decreased with the increasing concentration of UO_2_^2+^ under 364 nm excitation. And there was a good linear relationship between the UO_2_^2+^ concentration and the fluorescence intensity of the HTPAF fluorescent probe (y = 2613.05 − 45.63x, R^2^ = 0.998), where y represented the fluorescence intensity of the HTPAF fluorescent probe and x represented the concentration of uranyl ion. The fitted curves are shown in Figure 7b, and the fluorescence detection limit of HTPAF for uranyl ions was obtained as 76 nM (relative standard deviation <0.05% for both intra-day and inter-day), which was lower than the lowest concentration approved by US EPA in drinking water (0.126 μM) [45], by the formula LOD = 3δ/k [46], (where k represented the slope of the standard curve and δ represented the standard deviation of the fluorescence emission peak of the fluorescent probe HTPAF).

### 3.6. Binding Feature and Response Mechanism by DFT Calculations

**Figure 8:**
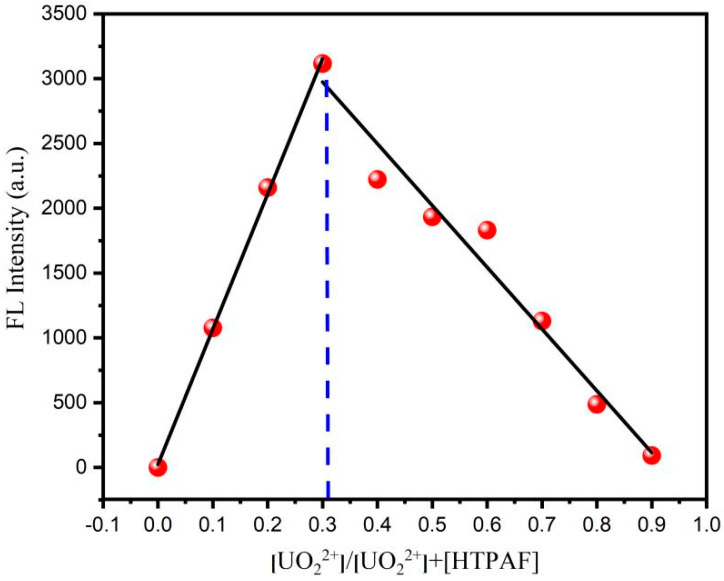
Job’s plot of HTPAF and UO_2_^2+^.

To investigate the binding characteristics of HTPAF to uranyl ions, Job’s plot was used to elucidate the chemomic attempts of the formed complexes (Figure 8). When [UO_2_^2+^]/([UO_2_^2+^] + [HTPAF]) was 0.3, the fluorescence intensity difference reached a maximum, indicating that the uranyl ion reached an optimal coordination ratio with HTPAF. Therefore, it could be tentatively judged that the coordination mode of the fluorescent probe HTPAF to UO_2_^2+^ was 2:1.

**Figure 9:**
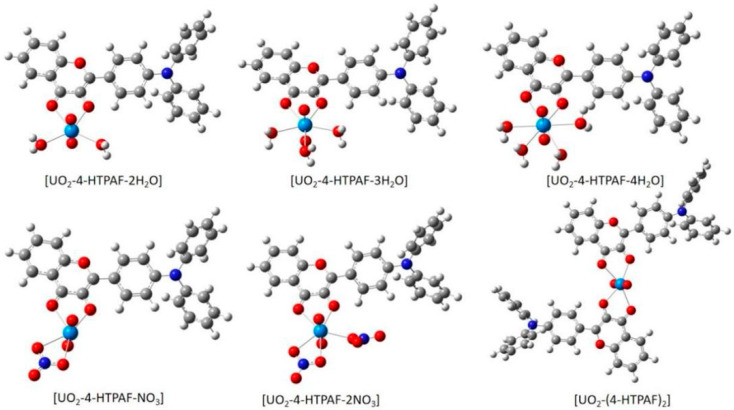
Possible coordination structures of uranyl with probe (HTPAF) and auxiliary ligands (H_2_O, NO_3_^−^) obtained by DFT calculation.

**Figure 10:**
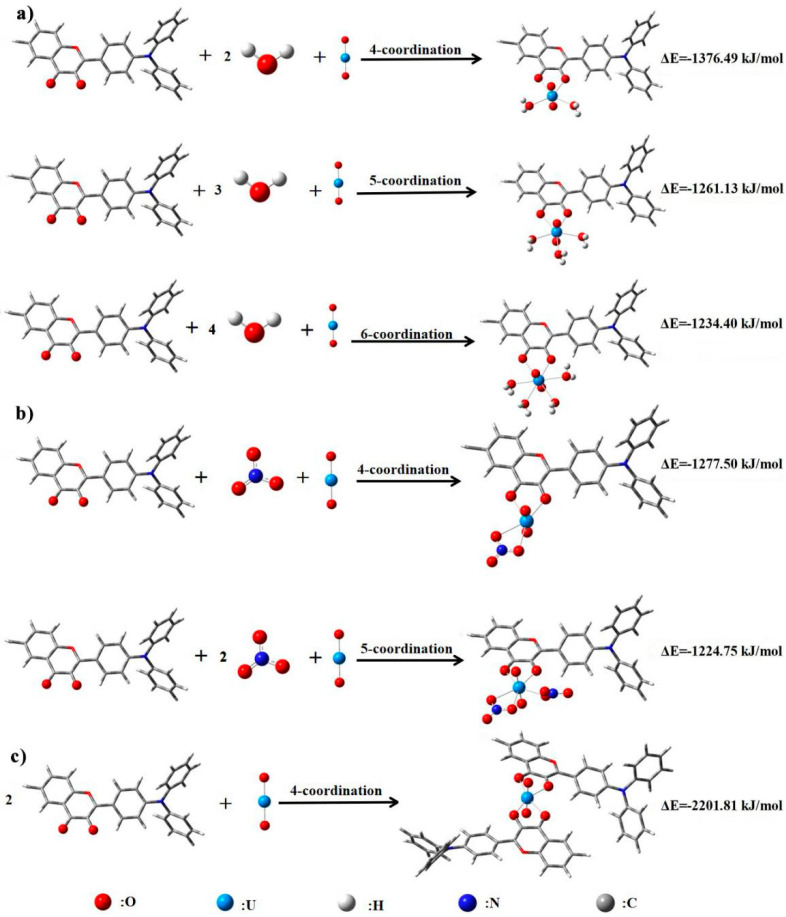
(**a**) Different coordination structures and binding energies between HTPAF and UO_2_^2+^ and H_2_O (**b**) Different coordination structures and binding energies between HTPAF and UO_2_^2+^ and NO_3_^−^ (**c**) HTPAF: UO_2_^2+^ = 2:1 forms a 4-coordination structure model and binding energy.

In general, uranyl ions form complexes with other organic substances that will form tetra-, pentacoordination, and hexacoordination, so that the conditions are for stable existence [47] (the two U=O that uranyl comes with are not considered). Moreover, the embodiment of the electric neutrality of the uranyl complex was due to the coordination with the anion present in the mixed test system, which had two types of auxiliary coordination groups (i.e., H_2_O and nitrate), where the nitrate came from the uranyl nitrate hexahydrate and the dilute nitric acid used to adjust the pH. Since the pH of the experimental system was 4.5 (when the fluorescent probe HTPAF started to dissociate), the hydrogen atoms on top of the fluorescent probe HTPAF were detached by default in the calculation. To study the coordination modes of uranyl complexes, density flooding theory calculations were implemented for all the formed coordination modes, and the structures of their coordination were [UO_2_-HTPAF-2H_2_O], [UO_2_-HTPAF-3H_2_O], [UO_2_-HTPAF-4H_2_O], [UO_2_-HTPAF-NO_3_], [UO_2_-HTPAF-2NO_3_], [UO_2_-(HTPAF)_2_], and the most probable coordination structures obtained by calculating the coordination structures are shown in Figure 9. Furthermore, their optimal coordination modes were also investigated by calculating their coordination binding energy (ΔE_bind_ for short) as shown in Table 1 and Figure 10. The ΔE_bind_ shown in the table was the energy difference possessed by the ligand before and after binding the uranyl ion. For example, for HTPAF+UO_2_^2+^ + 2H_2_O→[UO_2_-HTPAF-2H_2_O], ΔE_bind_ = E_coordination_ − E(UO_2_^2+^) − E(HTPAF) − E(2H_2_O). From Figure 10a,b, it can be found that the energy of ΔE_bind_ = −1376.49 kJ/mol for the 4-coordinated (H_2_O-assisted) coordination structure was smaller than that of the 4-coordinated (NO_3_^−^-assisted) coordination structure ΔE_bind_ = −1277.50 kJ/mol, which indicated that the water-assisted tetracoordinated structure was easier to form. In addition, if the two water molecules involved in the tetracoordination were replaced by one probe molecule, ΔE_bind_ decreased by 825.32 kJ/mol (Figure 10). From the analysis of the above results, it could be concluded that the coordination mode of HTPAF with UO_2_^2+^ was 2:1, which was consistent with our actual experimental results.

**Figure 11:**
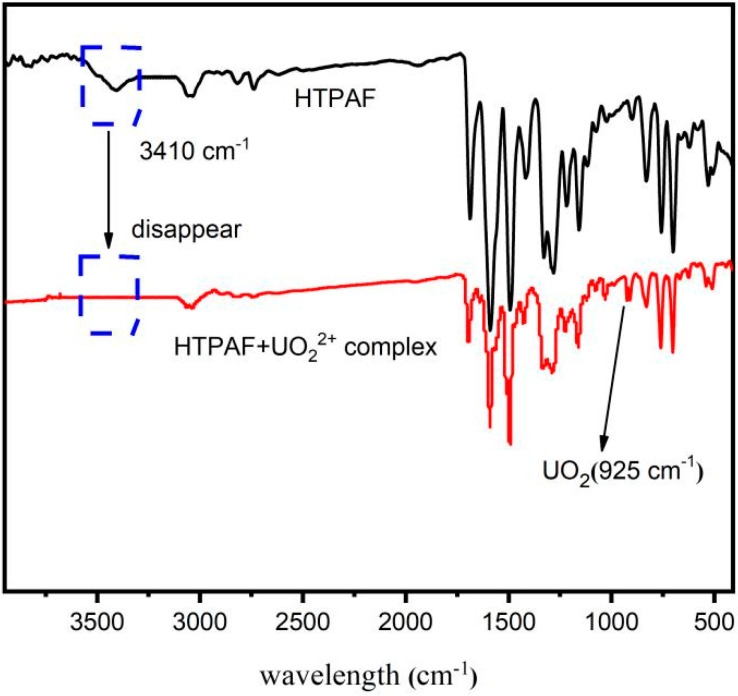
Comparison of infrared spectra before and after the combination of HTPAF and UO_2_^2+^.

**Figure 12:**
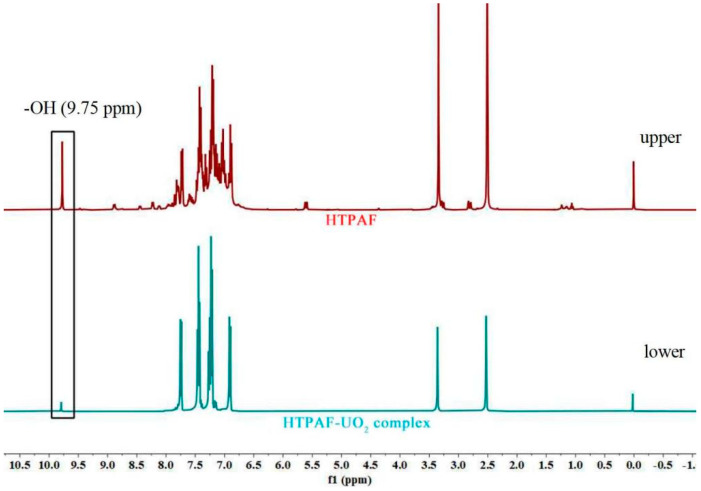
Comparison of ^1^HNMR spectra of fluorescent probe HTPAF before (upper) and after (lower) addition of uranyl ions.

**Figure 13:**
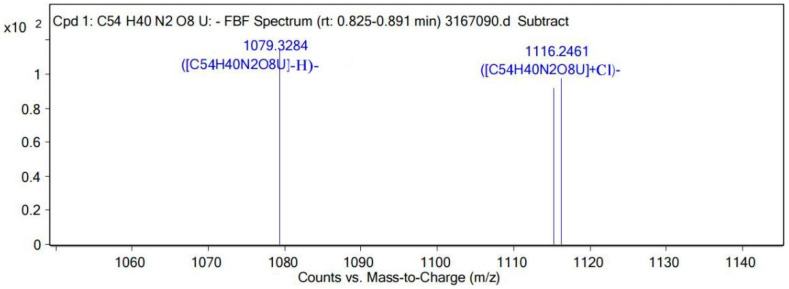
High-resolution mass spectrum of (HTPAF)_2_-UO_2_ complex.

In addition to theoretical calculations, the complexes were characterized accordingly by infrared spectroscopy, nuclear magnetic hydrogen spectroscopy (^1^HNMR) and high-resolution mass spectrometry (HRMS). Firstly, the IR spectra of the fluorescent probe HTPAF and the HTPAF-UO_2_ complex were compared, as shown in Figure 11. The disappearance of the 3410 cm^−1^ IR absorption peak in the IR spectrum of the HTPAF-UO_2_ complex compared to HTPAF confirmed that the -OH in the fluorescent probe HTPAF because of the coordination with uranyl. Furthermore, the IR spectrum of HTPAF-UO_2_ complex had an IR absorption peak at 925 cm^−1^ compared to HTPAF, which was clearly a stretching vibrational absorption peak of uranyl ion (920–930 cm^−1^) [13]. The comparison of the IR spectra of the HTPAF-UO_2_ complex with HTPAF demonstrated that HTPAF completed the coordination reaction with the UO_2_^2+^ ion. Secondly, the NMR hydrogen spectroscopy titration experiment further illustrated the coordination of HTPAF with UO_2_^2+^. As shown in Figure 12, the peak of hydroxyl hydrogen at 9.75 ppm shift of HTPAF disappeared when UO_2_^2+^ ions were added, indicating that -OH underwent the coordination reaction with UO_2_^2+^ ions. In addition, it is known from the high-resolution mass spectrometry (HRMS) analysis (Figure 13) that the peak 1079.3284 was consistent with the theoretical value of the complex [UO_2_+(HTPAF)_2_-H]^−^ (1081.93), which further confirmed the 2:1 coordination mode of HTPAF with UO_2_^2+^.

**Figure 14:**
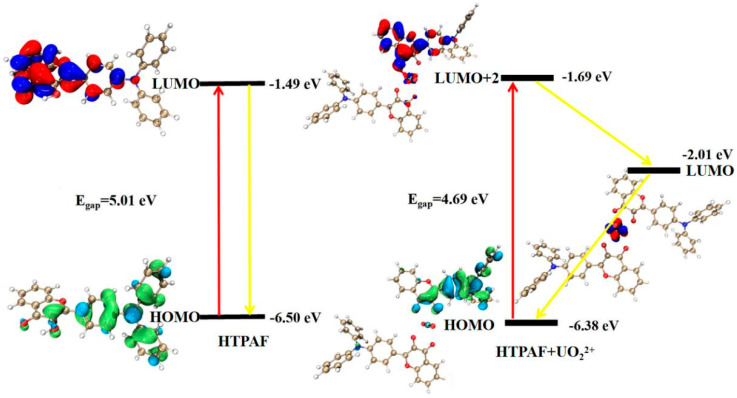
Schematic diagram and mechanism diagram of HTPAF fluorescent probe and fluorescent probe HTPAF forming complex with UO_2_^2+^.

**Scheme 3:**
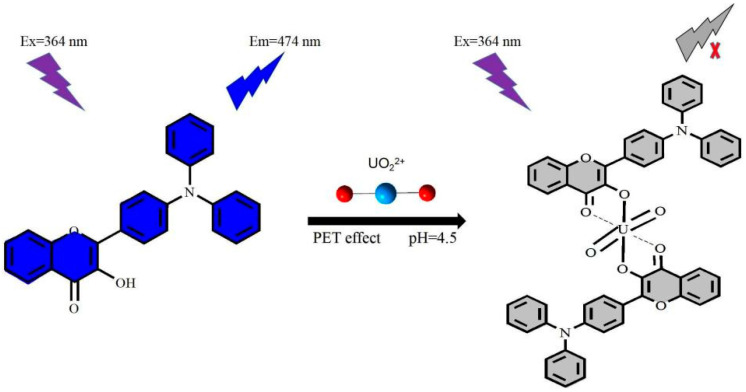
The mechanism for detection of UO_2_^2+^ ion by HTPAF.

In addition, the fluorescence recognition mechanism of HTPAF for UO_2_^2+^ was also simulated by DFT. The electrons in the LUMO orbitals mainly came from the HOMO excitation leap, and HOMO was the highest occupied molecular orbital of the fluorophore. As shown in Figure 14, after light excitation at 364 nm, electrons in the HOMO orbitals on the fluorescent probe HTPAF would leap to LUMO, and if no UO_2_^2+^ ions were bound, the electrons would return to the HOMO orbitals accompanied by energy release processes such as fluorescence emission. However, if bound to UO_2_^2+^, both the LUMO+2 and HOMO of the complexed coordination compound HTPAF +UO_2_^2+^ were mainly bound to the fluorescent probe HTPAF (Figure 14). The LUMO of HTPAF+UO_2_^2+^ was mainly confined to UO_2_^2+^, and after excitation, the electrons in the HOMO of HTPAF+UO_2_^2+^ leapt to the LUMO+2. Since the energy of the LUMO of HTPAF+UO_2_^2+^ (−2.01 ev) was lower than that of the LUMO+2 of HTPAF+UO_2_^2+^ (−1.69 ev), the electrons from LUMO+2 of HTPAF+UO_2_^2+^ could flow to the LUMO of HTPAF+UO_2_^2+^. i.e., the excited electrons on the LUMO of HTPAF+UO_2_^2+^ could not return to the ground state, which lead to the fluorescence burst. From the calculation results, it was obvious that it was a typical photoelectron transfer (PET) (Scheme 3).

### 3.7. Comparison of This Work with Other Reports

A comparison of the probe HTPAF with some UO_2_^2+^ ion probes was shown in Table 2. The test conditions of HTPAF were more favorable for application to real-world environments than other probes.

**Table 2:** Comparison of present work with the literature reports.

Ref.	Type	Test Conditions	Linear Range	LOD	Interfering Ions
[20]	Turn off	DMA:H_2_O (*v*/*v* = 95:5), pH = 6	10–150 nM	10 nM	No interference
[21]	Turn off	DMSO, pH = 4	1.7–490 μM	410 μM	Th^4+^, Al^3+^, Fe^3+^
[22]	Turn on	EtOH:H_2_O (*v*/*v* = 90:10), pH = 10.3	4.2–105 nM	0.84 nM	Cu^2+^
[17]	Turn off	THF:H_2_O (*v*/*v* = 5:95), pH = 4.5	1–10 μM	0.5 nM	Cu^2+^
[18]	Turn on	EtOH:H_2_O (*v*/*v* = 80:20), pH = 5.5	0–84 nM	2.1 nM	Al^3+^
[48]	Turn off	Toluene, pH (neutral)	0–3.3 μM	83.33 nM	No interference
[49]	Turn off	Aqueous solution, pH = 6	0–20 μM	1.7 nM	Al^3+^ (minor)
[50]	Turn off	Aqueous solution, pH = 5.5	1.12–121.1 nM	0.41 nM	No interference
[16]	Turn off	Aqueous solution, pH = 5.5	0–100 nM	2.14 nM	Th^4+^ (minor)
[51]	Turn off	DMF, pH = 6	0–10 μM	8.3 nM	No interference
This work	Turn off	DMSO:H_2_O (*v*/*v* = 5:95), pH = 4.5	0–50 μM	76 nM	No interference

## 4. Conclusions

In summary, a new flavonoid fluorescent probe HTPAF with triphenylamine functional group was designed and synthesized. The fluorescent probe HTPAF had a large Stokes shift (110 nm) and could selectively detect UO_2_^2+^ in high water ratio (DMSO:H_2_O, *v*/*v* = 5:95) and an acidic environment (pH = 4.5) without the presence of general metal ions and specific metal ions (e.g., Al^3+^,Cu^2+^, Fe^3+^, Th^4+^). In addition, Job’s plot, HRMS, FT-IR, ^1^HNMR experiments, and DFT calculations together demonstrated that the coordination mode of HTPAF with UO_2_^2+^ was 2:1, and the oxygen atom of -OH and the oxygen atom of the neighboring ketone (C=O) in flavonol were coordinated with UO_2_^2+^ to form a 4-ligand structure, resulting in a fluorescence intensity of HTPAF in the range of 0–50 μM with good linearity and a detection limit of 76 nM. Furthermore, due to the large Stokes shift and the absence of interference from other metal ions, the probe had potential applications for the detection of UO_2_^2+^ in the environment.

## Figures and Tables

**Table 1:** The binding energy of coordination compounds formed by HTPAF and UO_2_^2+^.

Structure of Coordination Compounds	Coordination Compounds (a.u.)	NO_3_^−^ (a.u.)	H_2_O (a.u.)	UO_2_^2+^ (a.u.)	HTPAF (a.u.)	ΔE_bind_ (kJ/mol)
HTPAF-UO_2_-2H_2_O	−4673.98	/	−152.87	−627.16	−3893.42	−1376.49
HTPAF-UO_2_-3H_2_O	−4750.38	/	−229.31	−627.16	−3893.42	−1261.13
HTPAF-UO_2_-4H_2_O	−4826.80	/	−305.75	−627.16	−3893.42	−1234.40
HTPAF-UO_2_-NO_3_	−4801.52	−280.45	/	−627.16	−3893.42	−1277.50
HTPAF-UO_2_-2NO_3_	−5081.96	−560.90	/	−627.16	−3893.42	−1224.75
(HTPAF)_2_-UO_2_	−8414.85	/	/	−627.16	−3893.42	−2201.81

## Supplementary Materials

The following supporting information can be downloaded at: https://www.mdpi.com/article/10.3390/s22186987/s1, Figure S1: The ^1^HNMR spectra of HTPAF; Figure S2: The ^13^CNMR spectra of HTPAF; Figure S3: The high-resolution mass spectrometry of HTPAF.
