# Supplementary material for: A Novel Triphenylamine-Based Flavonoid Fluorescent Probe with High Selectivity for Uranyl in Acid and High Water Systems
Source: Sensors (Basel). 2022 Sep 15;22(18):6987. doi: 10.3390/s22186987 (PMC9503699; doi:10.3390/s22186987)
Supplement: Supplementary file 1 [file sensors-22-06987-s001.zip › sensors-1886081-supplementary.pdf]

Supporting information

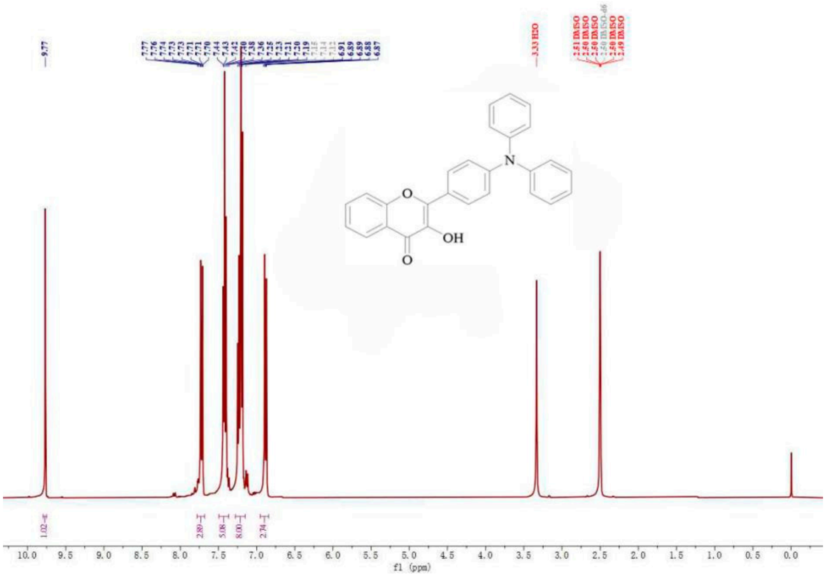

Figure S1. The ^{1}HNMR spectra of HTPAF.

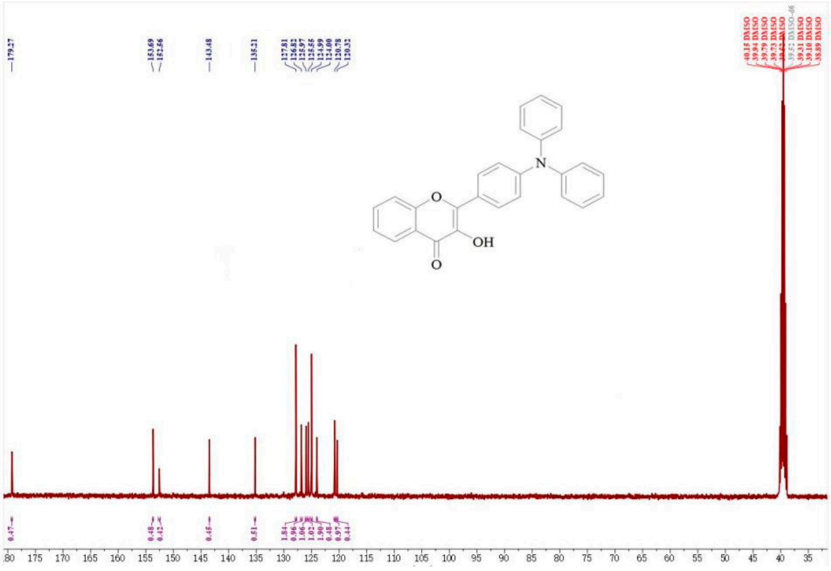

Figure S2. The ^{13}CNMR spectra of HTPAF.

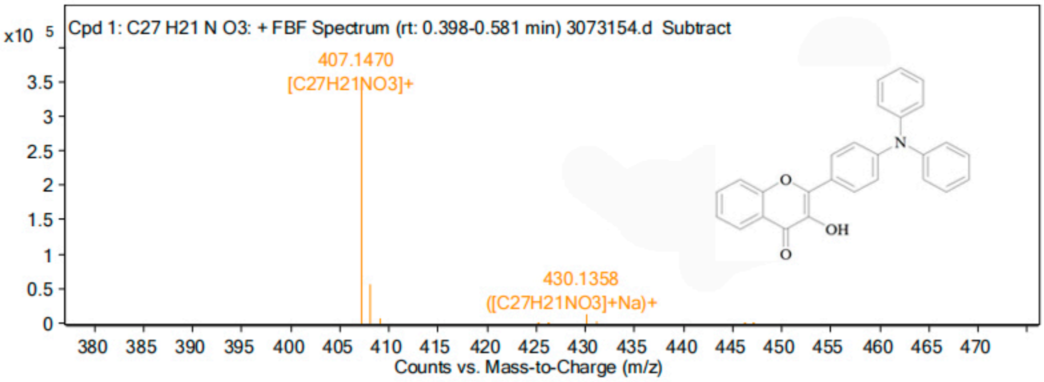

Figure S3. The high-resolution mass spectrometry of HTPAF.
